# Infiltration of Blood-Derived Macrophages Contributes to the Development of Diabetic Neuropathy

**DOI:** 10.1155/2019/7597382

**Published:** 2019-08-26

**Authors:** Jing-Jing Sun, Lin Tang, Xiao-Pei Zhao, Jun-Mei Xu, Yang Xiao, Hui Li

**Affiliations:** ^1^Department of Anesthesiology, The Second Xiangya Hospital, Central South University, 139 Ren-Min Road, Changsha 410011, China; ^2^Hunan Provincial Anesthesia Clinics and Technology Research Center, 139 Ren-Min Road, Changsha 410011, China; ^3^Department of Metabolism and Endocrinology, The Second Xiangya Hospital, Central South University, Changsha, China

## Abstract

**Background and Objective:**

Diabetic neuropathic pain (DNP) is a common complication associated with diabetes. Currently, its underlying pathomechanism remains unknown. Studies have revealed that the recruitment of blood monocyte-derived macrophages (MDMs) to the spinal cord plays a pivotal role in different models of central nervous system injury. Therefore, the present study aimed at exploring the infiltration and function of MDMs in DNP using a mice model.

**Methods:**

Diabetes was induced using streptozotocin in male A/J mice. Mechanical withdrawal thresholds were measured weekly to characterize neuropathy phenotype. Quantitative analysis of CD11b was performed and visualized by immunofluorescence. Spinal cord cells were isolated from myelin and debris by Percoll gradient. Flow cytometry was used to label CD11b and CD45 antibodies to differentiate MDMs (CD45^high^CD11b^+^) from resident microglia (CD45^low^CD11b^+^). Mice were injected with clodronate liposomes to investigate the role of MDMs in DNP. The successful depletion of monocytes was determined by flow cytometry.

**Results:**

The DNP mice model was successfully established. Compared with nondiabetic mice, diabetic mice displayed a markedly higher level of CD11b immunofluorescence in the spinal cord. The number of CD11b-positive microglia/macrophages gradually increased over the 28 days of testing after STZ injection, and a significant increase was observed on Day 14 (*P* < 0.01) and 28 (*P* < 0.01). Further analysis by flow cytometry showed that the infiltration of peripheral macrophages began to increase in 2 weeks (*P* < 0.001) and reached a maximum at 4 weeks (*P* < 0.001) post-STZ injection compared to the control. The depletion of MDMs by clodronate liposomes alleviated diabetes-induced tactile allodynia (*P* < 0.05) and reduced the infiltration of MDMs (*P* < 0.001) as well as the expression of IL-1*β* and TNF-*α* in the spinal cord (*P* < 0.05).

**Conclusions:**

The infiltration of blood MDMs in the spinal cord may promote the development of painful neuropathy in diabetes.

## 1. Introduction

Diabetic neuropathic pain (DNP) is defined as pain caused by abnormalities in the peripheral somatosensory system [1], occurring in nearly 40% of type 1 diabetic patients [2, 3]. However, the current therapy may be insufficient to combat allodynia due to a limited understanding of the cellular and molecular pathways [4].

It is well known that microglia are involved in the development of neuropathic pain after peripheral nerve injury [5]. However, it is still a subject of intense debate whether activated microglia under different pathological conditions are resident cells or monocyte-derived macrophages (MDMs) that are recruited from peripheral circulation [6, 7]. The previous understanding of the role of MDMs is limited due to the lack of markers or morphological characteristics to distinguish microglia and MDM. Recent work demonstrated that MDMs display different inflammatory profiles and function from microglia [8, 9]. MDMs in spinal cord promotes the hyperalgesia based on different models of chronic pain [10]. However, the role of MDMs in the development of diabetic neuropathy has not yet been clarified.

Using the monocyte-depletion approach, the present study aimed at characterizing the dynamic changes and the role of infiltrated MDMs in the spinal cord during the development of diabetic neuropathy.

## 2. Methods

### 2.1. Animals

All experiments were approved by the Hospital Ethics Committee of the Second Xiangya Hospital of Central South University and carried out in accordance with the National Institutes of Health guide for the care and use of laboratory animals (NIH Publications No. 8023, revised 1978). Methods and results are reported according to ARRIVE guidelines [11]. Seven-week-old male A/J mice were obtained from the Central South University Animal Services (Changsha, China) and were induced with diabetes at 8 weeks of age. All mice were housed in the Central South University Animal Services, had ad libitum access to food and water, and were maintained on a 12-hour light/dark cycle. All mice were sacrificed at 13 weeks of age.

### 2.2. Induction of Diabetes by STZ Injection

Eight-week-old male A/J mice were injected with STZ (Sigma-Aldrich, St. Louis, MO) to induce type 1 diabetes. Mice received low doses of STZ (40 mg/kg, intraperitoneal (i.p.) injection) for 5 consecutive days. Each injection was performed after 4 hours of fasting. Mice that did not reach hyperglycemia were excluded from the study.

Animal welfare (e.g., animal appearance and behavior) was assessed at least weekly by an animal care technician unaffiliated with the experimental team. During our experiments, 2 animals fulfilled predefined criteria for early termination of experiments (humane endpoints) when their body weights decreased above 20% after STZ treatment. The animals were euthanized. The replacement of animals was done after consultation with the Animal Care and Use Committee. All other animals survived to the end of the experiment, and welfare assessment showed no abnormalities concerning appearance or behavior at any time point.

### 2.3. Blood Glucose Measurements

Weight and blood glucose measurements (glucose diagnostic reagents; Sigma-Aldrich, St. Louis, MO) were collected one week after the initial injection and every week thereafter. Mice were fasted for 3 hours prior to the collection of blood from the tail. Mice were considered diabetic when blood glucose levels were >250 mg/dL.

### 2.4. Determination of Plasma Insulin, HbA1C, and Leukocytes

Plasma insulin and glycated hemoglobin (HbA1C) were analyzed by using ELISA kits (Beijing BHKT Clinical Reagent Co. Ltd., Beijing, China) as per the manufacturer's instruction. Blood was collected in EDTA-2K tubes. The neutrophil count and monocyte count were analyzed by automatic cell analyzers (ARCHITECT c8000, Abbott Corporation, Chicago, United States).

### 2.5. Monocyte Depletion

After the successful induction of diabetes via STZ injection, diabetic mice were i.p. injected with 200 *μ*L of clodronate liposomes or an equal volume of empty liposomes (FormuMax Scientific Inc., California, 7 mg/mL) followed by 100 *μ*L/20-25 g every three days thereafter. This protocol ensures effective monocyte depletion from the time of diabetic development until they are sacrificed.

### 2.6. Mechanical Threshold Test

The paw mechanical threshold test was conducted as described previously with slight modification [12]. In brief, mice were acclimated to the behavior facility and were put into the test facility for at least 30 minutes prior to the behavior test. The plantar surface of each hind paw was assessed at the indicated time point after STZ injection by von Frey hairs with logarithmically increasing stiffness (0.02–2.56 g; Stoelting Co., Wood Dale, IL), presented perpendicular to the plantar surface (2–3 seconds for each hair). The mean mechanical withdrawal threshold of three applications to each hind paw was calculated for each testing session. The group means were calculated, and the mice were sacrificed after behavioral testing at 6 weeks post-STZ.

### 2.7. Immunofluorescence

Mice were deeply anesthetized by sevoflurane and perfused with 4% paraformaldehyde for immunofluorescence assays. Immunofluorescences were undertaken using anti-rabbit CD11b (Affinity, OH, US, diluted in 1 : 400) [13]. The secondary antibody was FITC-conjugated Affinipure Goat Anti-Rabbit IgG (Proteintech, IL, diluted in 1 : 400). Immunofluorescence sections were cover-slipped with mounting medium (Vector Laboratories Inc., Burlingame, CA) and visualized by a fluorescence optical microscope. All images were analyzed with ImageJ software (National Institutes of Health) for automated cell counting. The analysis was done by an investigator who was blinded to the experimental groups.

### 2.8. Isolation of Spinal Cord Cells and Flow Cytometric Analysis

Mice were killed by an overdose of sevoflurane, and their spinal cords were harvested for flow cytometric analysis through the perfusion with PBS. A spinal cord lumbar vertebra was cut from individual mice, and cells were filtered through a 40 *μ*m nylon cell strainer (BD Biosciences). An isolation method by Percoll gradient (GE Healthcare; 70% and 30%) was used to separate cells from myelin and debris. The cells were gently collected from the interface of the gradient and washed for further flow cytometric analysis. The cells were then treated with purified rat anti-mouse CD16/32 (Fc Block; BD Pharmingen, 1 : 100) for 30 min in ice. After that, the cells were incubated with the following antibodies or the respective isotype controls for 20 min on ice: PE-cy7-labeled rat anti-mouse CD11b (BioLegend, 1 : 100) and FITC-labeled rat anti-mouse CD45 (BioLegend, 1 : 100). The cells were finally analyzed on the FACSCalibur cytometer (BD Biosciences) using the CellQuest software (BD Biosciences).

To determine the effective monocyte depletion, mice were euthanized by isoflurane overdose. Blood was collected by cardiac puncture, and mice were then perfused with PBS, followed by decapitation. Erythrocytes were lysed with ammonium chloride. Leukocyte single-cell suspensions were analyzed using the following antibodies: PE-cy7-labeled rat anti-mouse CD11b (BioLegend, 1 : 100) and PE-labeled rat anti-mouse F4/80 (BioLegend, 1 : 100). The cells were finally analyzed on the FACSCalibur cytometer (BD Biosciences) using the CellQuest software (BD Biosciences).

### 2.9. Proinflammatory Cytokines in the Spinal Cord

Spinal cord was harvested and weighed sections were homogenized in homogenization buffer. Samples were cold centrifuged and supernatant was used for the examination of IL-1*β* and TNF-*α* concentration levels using the quantitative sandwich enzyme immunoassay according to the manufacturer's instructions (R&D Systems, MN, USA). The levels of IL-1*β* and TNF-*α* were determined by comparing samples to the standard curve generated from the respective kits at 450 nm and were expressed as pg per mg tissue (spinal cord).

### 2.10. Statistical Analysis

Two-way repeated measures analysis of variance (2-way RM-ANOVA) with Fisher's least significant difference (LSD) posttest analyses were performed, as denoted in the manuscript. Group comparisons for microglia/macrophage immunoreactivity and number of MDMs were performed using the Mann-Whitney *U* test. All data are presented as mean ± SD. IBM SPSS Statistics Software version 20 (IBM, San Francisco, CA) was used for all statistical analyses, and a *P* value less than 0.05 was considered to be statistically significant.

## 3. Result

### 3.1. Development of Diabetic Neuropathy by STZ Injection

**Figure 1:**
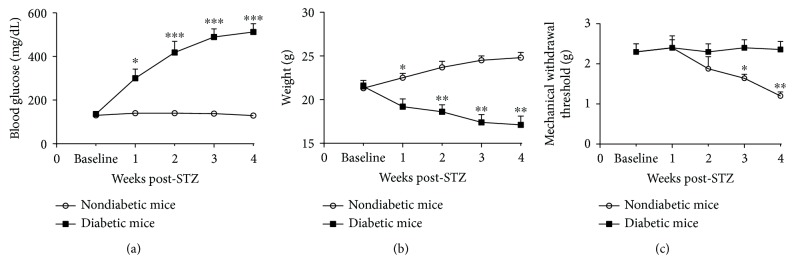
Development of hyperglycemia and mechanical allodynia in diabetic mice. (a) Blood glucose of diabetic mice increased significantly at each time point 1 week after STZ injection. (b) Diabetic mice did not gain weight, in contrast to their nondiabetic counterparts. (c) The mechanical withdrawal threshold of diabetic mice started decreasing significantly 2 weeks after STZ injection and persisted throughout the study period (^∗^*P* < 0.05, ^∗∗^*P* < 0.01, and ^∗∗∗^*P* < 0.001 vs. nondiabetic mice at all time points; 2-way RM-ANOVA and LSD posttest). *n* = 25‐30 mice per group.

A type 1 diabetic mouse model was established by the *i.p.* administration of STZ. As shown in Figure 1, treatment with STZ resulted in a significant increase of fasting blood glucose levels (512 ± 38 mg/dL in STZ-treated mice versus 132 ± 10 mg/dL in control mice; *P* < 0.001) (Figure 1(a)). The diabetic mice had a significantly lower body weight compared to nondiabetic mice (Figure 1(b)).

Both STZ-induced mice and nondiabetic mice received a weekly behavioral test to analyze mechanical sensitivity in the hind paw (Figure 1). Compared to nondiabetic mice, the diabetic mice displayed mechanical hypersensitivity to von Frey monofilaments at week 3 post-STZ treatment (*P* < 0.05). Mechanical allodynia reached maximum levels at four weeks and remained consistently high at five weeks post-STZ injection.

### 3.2. Infiltration of Blood-Derived Monocytes in the Spinal Cord Caused by Hyperglycemia

**Figure 2:**
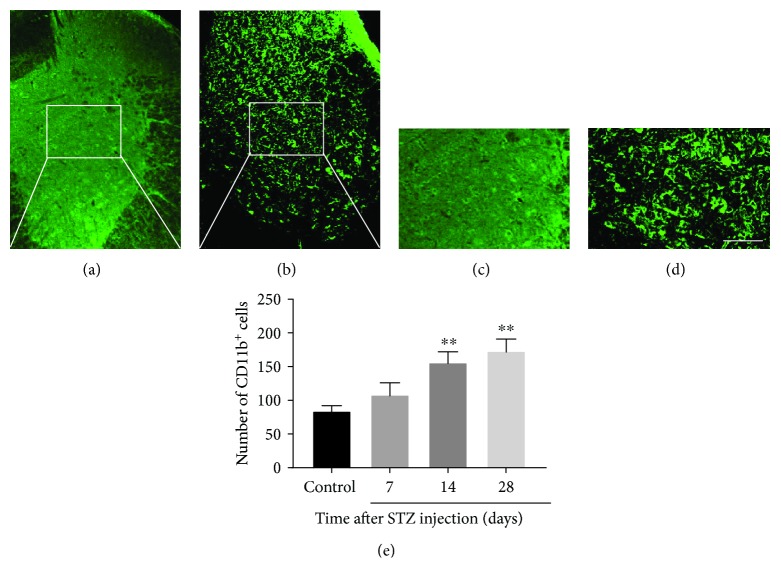
Hyperglycemia increases the microglia/macrophage activation in the spinal cord. (a–d) Photomicrographs show immunofluorescence of the microglia/macrophage marker CD11b in the spinal cord after STZ injection. Scale bar = 100 *μ*m for (a) and (b), 200 *μ*m for (c) and (d). (e) Quantification of microglia/macrophage immunoreactivity showing a significant increase in CD11b expression during 4 weeks. Numbers of animals: control, *n* = 6; D7, *n* = 6; D14, *n* = 6; D28, *n* = 6. Mann-Whitney *U* test was used. Data are means SEMs; ^∗∗^*P* < 0.01 vs. control.

Previous studies have reported the increased activation of microglia/macrophages in the spinal cord of the diabetic rat [14]. In the present study, STZ-treated diabetic mice displayed a significantly high CD11b^+^ immunofluorescence in the spinal cord, as compared with nondiabetic mice (Figure 2). This activation was maintained for at least 4 weeks after STZ injection. Moreover, the number of CD11b^+^ cells greatly increased at 2 weeks post-STZ injection compared to the control group (*P* < 0.01). These results suggest that diabetes induced the activation of microglia/macrophages in the spinal cord.

**Figure 3:**
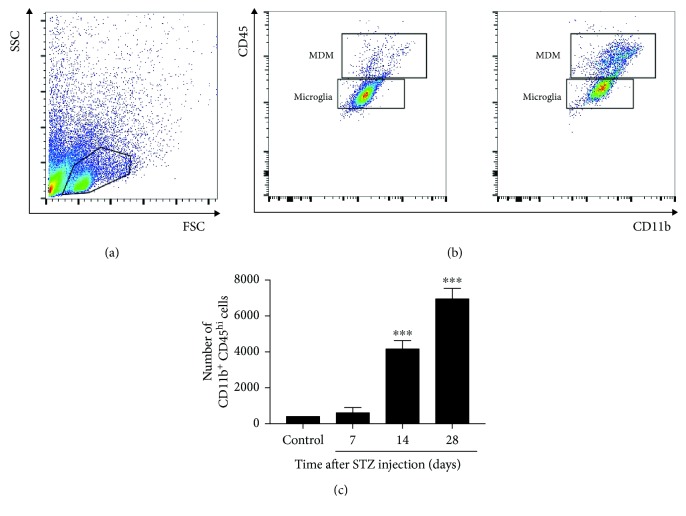
Hyperglycemia increases the infiltration of blood-derived macrophages in the spinal cord. (a, b) Examples of flow cytometry analysis of the spinal cord are shown, identifying monocyte-derived macrophages (MDMs) and microglia as CD45^+^CD11b^high^ and CD45^+^CD11b^low^, respectively. (c) Spontaneous infiltration of circulating monocytes to the spinal cord increases after STZ injection. Numbers of animals: control, *n* = 9; D7, *n* = 9; D14, *n* = 9; D28, *n* = 9. Mann-Whitney *U* test was used. Data are means SEMs; ^∗∗∗^*P* < 0.001 vs. control.

On a microscopic level, the infiltrated MDMs and resident microglia are indistinguishable due to a lack of the appropriate surface markers. To determine the infiltration of MDMs in the spinal cord following the induction of diabetes, flow cytometry was used to label CD11b and CD45 antibodies to differentiate MDMs (CD45^high^CD11b^+^) from resident microglia (CD45^low^CD11b^+^) based on CD45 expression [15, 16]. As shown in Figure 3, the infiltration of peripheral macrophages significantly increased at 2 weeks post-STZ injection compared to the control (*P* < 0.001). A representative example of CD45 and CD11b double-labeled cells in Figure 3(a) and Figure 3(b) shows that in the spinal cord, 38.8% of the CD11b^+^ cells were CD45^high^CD11b^+^ and 58.9% CD11b^+^ cells were CD45^low^CD11b^+^ at 4 weeks following STZ-injection.

### 3.3. Depletion of MDMs Relieved Diabetic Neuropathy

**Figure 4:**
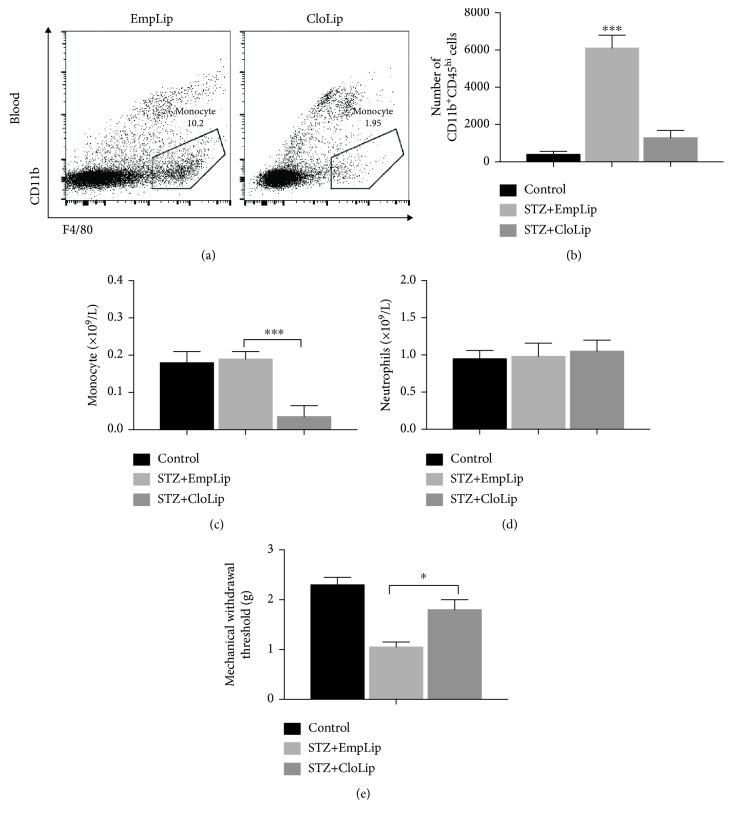
Alleviation of diabetes-induced tactile allodynia and infiltration of blood MDMs by clodronate liposomes. (a) Flow cytometric analysis showing successful depletion of monocytes in peripheral blood after treatment of clodronate liposomes. (b) Infiltration of blood-derived macrophages in the spinal cord decreased in diabetic mice injected with clodronate liposomes as compared with empty liposomes. (c) Treatment with clodronate liposomes reduced monocytes but not neutrophils (d) in blood. (e) The mechanical withdrawal thresholds in control (a group of age-matched nondiabetic control mice) and STZ-induced diabetic mice after the injection of the empty liposomes or clodronate liposomes. *n* = 6‐8 mice per group. One-way ANOVA and LSD posttest were used. Data are means SEMs; ^∗^*P* < 0.05 and ^∗∗∗^*P* < 0.001 vs. control.

**Figure 5:**
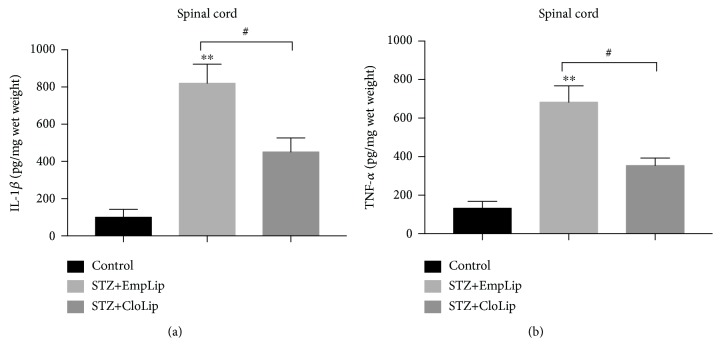
Changes in the expression of IL-1*β* (a) and TNF-*α* (b) of experimental groups in the spinal cord after liposome treatment. *n* = 6‐8 mice per group. One-way ANOVA and LSD posttest were used. Data are means SEMs; ^∗∗^*P* < 0.01 vs. control. ^#^*P* < 0.05.

To determine whether the increased infiltration of MDMs plays a role in diabetic neuropathy, clodronate liposomes were used to deplete monocytes. The treatment of clodronate liposome had no significant effect on blood glucose, serum insulin, HbA1C, and body weights (Table 1) but greatly decreased the infiltration of blood-derived macrophages as well as the expression of IL-1*β* and TNF-*α* in the spinal cord in diabetic mice (Figure 4(b) and Figure 5). Flow cytometric analysis of peripheral blood confirmed monocyte depletion in clodronate liposome-treated mice (Figure 4(a)). Treatment with clodronate liposomes reduced monocytes but not neutrophils in blood (Figures 4(c) and 4(d)) (*P* < 0.001). The mechanical allodynia in diabetic mice was also abolished by clodronate liposome administration (Figure 4(e)) (*P* < 0.05).

## 4. Discussion

The specific role of MDMs in the spinal cord in diabetic neuropathy is largely unknown due to difficulties in differentiating the resident microglia from infiltrated macrophages using the traditional histochemical analysis. Here, it was shown for the first time that numerous MDMs infiltrated into the spinal cord throughout the development of diabetic neuropathy, and their deletion alleviated diabetes-induced tactile allodynia. These findings suggest that the infiltrated peripheral MDMs contribute to mechanical allodynia in response to the painful neuropathy in diabetes.

Microglia/macrophages in the spinal cord, one of the most susceptible sensors upon injury, have been reported to contribute to the development of DNP recently [14, 17–19]. In DNP mice, the microglia/macrophages are activated and undergo amoeboid hypertrophic changes. The administration of the selective microglia inhibitor minocycline alleviates thermal and mechanical hypersensitivity in early diabetic pain neuropathy [20]. Moreover, Cheng et al. reported that the activation of microglia/macrophages was present early in the spinal cord and was positively correlated with mechanical allodynia induced by STZ injection-induced diabetic rats [21]. In the present study, STZ i.p. administration rendered the persistent increase of blood glucose level suggesting the successful establishment of diabetic mice. The pain threshold decreased starting at week 2 post-STZ injection indicating the development of painful neuropathy. Consistent with the previous studies, CD11b positive staining was also increased in the spinal cord suggesting a global activation of microglia/macrophages in the spinal cord.

It is well known that the peripheral infiltrated macrophages and activated resident microglia can be differentiated by CD45^high^CD11b^+^ (for MDM) and CD45^low^CD11b^+^ staining in the FACs assay [15, 16, 22]. A recent study by Stranahan et al. found that infiltrated CD45^high^CD11b^+^ MDM cells in the brain in leptin receptor mutant (db/db) mice and the blockage of blood-brain barrier breakdown by the protein kinase C *β* inhibitor reversed the number of blood-derived macrophages [23]. However, the infiltration and function of MDM in the spinal cord of mice with diabetic neuropathy remain unclear. In the present study, the population of MDMs took around 38.8% of the CD11b^+^ leukocytes in the spinal cord in the DNP mice. These findings suggest that a large population of MDMs infiltrated into the spinal cord. However, the role of the infiltrated MDMs in DNP is still unknown.

The functions of the infiltrated MDMs may vary in different models of central nervous system (CNS) injury. For example, blood MDMs exert an anti-inflammatory function in recovery in spinal cord injury [24]. In contrast, some studies have shown that these cells initiate secondary injury in models of ischemic stroke and traumatic brain injury [25, 26]. Notably, in CNS injury, the MDMs directly infiltrate into the local injured zone, likely together with other inflammatory cells such as neutrophils or immune cells. Peripheral neuropathy-related inflammation may be different from CNS injury since the spinal cord is not subjected to direct damage. Thus, the infiltrated MDMs may also have distinct functions from those in CNS injury. Previous studies have shown that clodronate liposome-mediated depletion of MDMs prolonged the hyperalgesic response to intraplantar IL-1*β* injection [27]. Monocyte depletion also delayed the resolution of carrageenan-induced thermal and mechanical hyperalgesia [27]. These findings suggested that blood MDMs may exert antihyperalgesia effects in the inflammatory pain. In the present study, clodronate liposome injection greatly cleared the population of monocytes as indicated by a FACs assay. Interestingly, the depletion of the monocytes greatly attenuated the development of mechanical allodynia in the DNP mice, without significantly affecting systemic characteristics including blood glucose, serum insulin, HbA1C, and body weights. These results strongly indicated that the infiltrated MDMs exacerbated the diabetic neuropathy induced by STZ injection. These results also imply that persistent hyperglycemia not only leads to the damage of the peripheral nerves but may also affect the central nervous system. This assumption is supported by the activated CD11b positive staining, the increased number of MDMs, and the expression of IL-1*β* and TNF-*α* in the spinal cord from diabetic mice. However, the exact functions of MDMs in the spinal cord require further studies in the future.

There are several concerns in the present study. Firstly, the reduction of body weight in the DNP may be a confounding factor for pain behavior. A recent clinical study shows that abnormal body weight (underweight and obese) decreased the pain threshold [28]. These findings suggest that the reduction of body weight may contribute to pain hypersensitivity. However, in the present study, monocyte depletion did not greatly affect the body weight but inhibited pain hypersensitivity in the diabetic mice. These findings argue that monocyte activation, but not body weight, mediates pain hypersensitivity induced by hyperglycemia. Secondly, recent studies have shown that diabetic mice have a defect in the mobilization of stem/progenitor cells after directly stimulating bone marrow with growth factors [29]. Diabetes may also impair the interactions between long-term hematopoietic stem cells and osteopontin-positive cells in the endosteal niche of mouse bone marrow [30]. However, no direct evidence demonstrates the impact of hyperglycemia on hemopoietic stem cell proliferation and differentiation. Furthermore, diabetic patients do not suffer from an excess risk of hematological malignancies. All these findings strongly indicate that the effect of diabetes on MDM activation and infiltration is not through its impact on the hemopoietic stem cell proliferation and differentiation.

## 5. Conclusions

It was proposed that peripheral infiltration of MDMs in the spinal cord increased over time during the development of diabetic neuropathy and may work in concert with resident microglia towards the etiopathology of painful diabetic neuropathy.

## Figures and Tables

**Table 1:** Body weights and blood concentration of experimental groups after liposome treatment.

Experimental groups	Body weight (g)	Blood glucose concentration (mg/dL)	Serum insulin (pmol/L)	HbA1C
Control	24.3 ± 4.8	115.8 ± 28.5	574.6 ± 35.2	4.9 ± 0.2
STZ+empty liposomes	16.1±3.3^∗∗^	485.5±46.3^∗∗∗^	12.2±2.7^∗∗∗^	8.1±0.3^∗∗∗^
STZ+clodronate liposomes	16.8±3.6^∗∗^	401.6±45.8^∗∗∗^	12.8±2.1^∗∗∗^	7.9±0.2^∗∗∗^

Mean values ± standard error of the mean (S.E.M.). STZ: streptozotocin. *n* = 6‐8 mice per group. One-way ANOVA and LSD posttest were used. ^∗∗^*P* < 0.01 and ^∗∗∗^*P* < 0.001 vs. control.

## Data Availability

The data used to support the findings of this study are available from the corresponding author upon request.
